# 843. Systematic Review of Two Dermal Matrices: Scar Quality, Grafting Time, and Infection Rates

**DOI:** 10.1093/jbcr/irag033.282

**Published:** 2026-04-06

**Authors:** Rohit Mittal, Claire Witherel, Steven A Kahn, Roselle E Crombie

**Affiliations:** South Carolina Burn Center at Medical University of South Carolina, Charleston, SC; Integra LifeSciences, Metuchen, New Jersey; Medical University of South Carolina, Charleston, SC; Connecticut Burn Center/Yale New Haven Health, Westport, CT

## Abstract

**Introduction:**

Dermal matrices are valuable tools for reconstructing complex soft tissue wounds after burn/traumatic injury, infection, or planned surgery. Practice patterns and matrix selection vary based on surgeon preference, experience, and institutional resources, with no clear consensus on optimal clinical use. Two dermal matrices—a bilayer collagen/glycosaminoglycan-silicone matrix (bioengineered from xenograft material) and a bilayer polyurethane-silicone matrix (completely synthetic)—represent different technologies yet are used in similar clinical scenarios, including full-thickness burns. This systematic study evaluated differences in time to skin grafting, infection rate, and long-term scar quality between these matrices.

**Methods:**

A systematic PubMed literature review evaluated two dermal matrices using search queries: "bilayer dermal regeneration template (IDRT)" AND ("Vancouver Scar Scale (VSS)" OR "time to graft" OR "infection rate"), and "biodegradable polyurethane matrix (BTM)" AND ("Vancouver Scar Scale (VSS)" OR "time to graft" OR "infection rate"). Inclusion criteria: ≥3 patients and > 6-month follow-up for VSS. Average values for each metric were analyzed via unpaired t-test with Welch's correction.

**Results:**

The search identified 2979 IDRT and 431 BTM articles; 34 contained relevant outcome data. VSS did not differ between treatments (IDRT: 3.383 ± 1.995, n = 6; BTM: 7.390 ± 3.847, n = 3; *p*=.2045). IDRT showed significantly shorter time to graft (16.77 ± 6.3 vs 34.95 ± 4.453 days, *p*=.0003) and lower infection rate (12.52 ± 3.136% vs 17.4 ± 2.68%, *p*=.0301).

**Conclusions:**

This systematic review demonstrates significant short-term outcome differences between these dermal matrices. IDRT showed shorter grafting time and lower infection rates versus BTM, while VSS scores were comparable. These retrospective benchmarks are important given the absence of randomized prospective studies. Future validation against multi-center databases (National Burn Repository, Burn Care Quality Platform) is planned.

**Applicability of Research to Practice:**

This work highlights critical need for standardized outcome reporting in dermal matrix studies, as only 34 of 3410 articles met inclusion criteria, demonstrating significant reporting heterogeneity that impedes meta-analysis and evidence synthesis.

**Funding for the study:**

This study was funded by industry support from a medical device manufacturer.

